# Necrology

**Published:** 1902-04

**Authors:** 


### NECROLOGY.

Dr. C. J. Carrick, of Pittsburg, Pa., a graduate of the Royal College of Veterinary Surgeons, died the latter part of February.

At Charleston, Ill., in March, Tarlton C. Miles, better known as "Farmer" Miles. Born near Frankfort, Ky., in 1825, he had reached the age of seventy-five years. He was one of the most successful operators on cryptorchids in this country. For many years he gave instructions to large bodies of students on his methods of operating on ridgelings. He had travelled well over the English-speaking world, operating before many of the most prominent European veterinarians. His two sons—one a graduate of the Montreal Veterinary College, the other a graduate of the Chicago Veterinary College—survive him.

